# Medical Societies

**Published:** 1904-11

**Authors:** 


### Society Notes.

### Medical Societies.

THE TRI-STATE MEDICAL SOCIETY of Arkansas, Louisiana and Texas was organized at Shreveport, La., October 14th ult. Dr. J. R. Dale, of Texarkana, was elected President.

SAN ANGELO DISTRICT MEDICAL SOCIETY held its semi-annual meeting (District No. 4) at San Angelo October 18th and 19th ult. A. S. Love, President, Ballinger, Texas; S. C. Parsons, San Angelo, Secretary. No report received.

THE FAMOUS BRAZOS VALLEY MEDICAL SOCIETY will hold its eighteenth semi-annual meeting at Franklin, Texas, November 22d and 23d inst. As usual, they have a fine prorgam. Dr. E. N. Shaw, Cameron, President, and the irrepressible Dr. W. B. Briggs—a Confed. Vet.—is Secretary, for life.

THE NOLAN-FISHER-STONEWALL COUNTY MEDICAL SOCIETY will meet next time at Roby, Fisher county, first Tuesday in December (prox.). Dr. J. G. Hanbright will write on "Tuberculosis"; Dr. Scott on "Puerperal Sepsis," and Dr. McCamant on "Pneumonia." Dr. L. B. Roebuck, Sweetwater, is Secretary.

THE SOUTHWESTERN TRI-STATE MEDICAL SOCIETY had a large and interesting meeting at Dallas November 4th and 5th. Dr. Tupper, of St. Louis, was the guest of honor, and made a fine impression. After labors there was a smoker, largely attended. Toasts, sandwiches and beer and spicy "responses" were the order, and all were much enjoyed. We had the pleasure of being present by invitation, and anted our chip, of course. Meets next at Oklahoma City.

